# Editorial Comment: What Adults Teach Urologists About Hypospadias?

**DOI:** 10.1590/S1677-5538.IBJU.2024.9910

**Published:** 2024-03-20

**Authors:** Luciano A. Favorito

**Affiliations:** 1 Universidade do Estado do Rio de Janeiro Unidade de Pesquisa Urogenital Rio de Janeiro RJ Brasil Unidade de Pesquisa Urogenital - Universidade do Estado do Rio de Janeiro - Uerj, Rio de Janeiro, RJ, Brasil; 2 Hospital Federal da Lagoa Serviço de Urologia Rio de Janeiro RJ Brasil Serviço de Urologia, Hospital Federal da Lagoa, Rio de Janeiro, RJ, Brasil

**Warren Snodgrass**^1^, **Nicol Bush**^1^

**Urol Clin North Am. 2023 Aug;50(3):447-453.**

DOI: 10.1016/j.ucl.2023.04.005 |ACCESS:3738570

## COMMENT

In this interesting review, Prof. Snodgrass (1) shows important points of technique of the hypospadias repair. Is important to know that men with hypospadias who have no glans fusion have increased risk for urine spraying. The implication of hypospadias in adults are very important too. As many as one of every three men with uncorrected distal hypospadias have penile curvature which can impact sexual function and the hypospadias repair can be done in adults with the same expected outcomes as in boys, but the overall experience for them is more traumatic. The consequences of uncorrected, or unsuccessfully corrected, hypospadias can impact a man's well-being throughout life. The authors concluded that the birth defects of the penis are best corrected in childhood, the goal of surgery is to make the abnormal anatomy normal to best ensure normal urinary and sexual function, normal esthetics are also important to patients born with hypospadias, meaning surgeons should avoid incisions outside the median raphe and circumcision lines, use only penile skin to cover the penis, and avoid glanular epithelial stitches which can leave scars and divots, successful repair making the penis straight with a normal neomeatus and symmetric circumferential coverage with penile skin has little risk for future complications and adults who present with uncorrected hypospadias, or complications after prior repairs, can still have successful surgery that includes making a normal neomeatus. These lessons that adult patients teach urologists about hypospadias can be summarized by the conclusion that boys born with this birth defect want to grow into men with a normal penis.

This paper is very important to reconstructive urology.
